# Inhibition of the processing of miR-25 by HIPK2-Phosphorylated-MeCP2 induces NOX4 in early diabetic nephropathy

**DOI:** 10.1038/srep38789

**Published:** 2016-12-12

**Authors:** Hyung Jung Oh, Mitsuo Kato, Supriya Deshpande, Erli Zhang, Das Sadhan, Linda Lanting, Mei Wang, Rama Natarajan

**Affiliations:** 1 Department of Diabetes Complications and Metabolism, Beckman Research Institute of City of Hope, Duarte, California, USA; 2 Ewha Institute of Convergence Medicine, Ewha Womans University Mokdong Hospital, South Korea; 3 Tsingua University, Beijing, China

## Abstract

Phosphorylated methyl-CpG binding protein2 (p-MeCP2) suppresses the processing of several microRNAs (miRNAs). Homeo-domain interacting protein kinase2 (HIPK2) phosphorylates MeCP2, a known transcriptional repressor. However, it is not known if MeCP2 and HIPK2 are involved in processing of miRNAs implicated in diabetic nephropathy. p-MeCP2 and HIPK2 levels were significantly increased, but Seven in Absentia Homolog1 (SIAH1), which mediates proteasomal degradation of HIPK2, was decreased in the glomeruli of streptozotocin injected diabetic mice. Among several miRNAs, miR-25 and its precursor were significantly decreased in diabetic mice, whereas primary miR-25 levels were significantly increased. NADPH oxidase4 (NOX4), a target of miR-25, was significantly increased in diabetic mice. Protein levels of p-MeCP2, HIPK2, and NOX4 were increased in high glucose (HG)- or TGF-β-treated mouse glomerular mesangial cells (MMCs). miR-25 (primary, precursor, and mature) and mRNA levels of genes indicated in the *in vivo* study showed similar trends of regulation in MMCs treated with HG or TGF-β. The HG- or TGF-β-induced upregulation of p-MeCP2, NOX4 and primary miR-25, but downregulation of precursor and mature miR-25, were attenuated by *Hipk2* siRNA. These results demonstrate a novel role for the SIAH1/HIPK2/MeCP2 axis in suppressing miR-25 processing and thereby upregulating NOX4 in early diabetic nephropathy.

Diabetic nephropathy (DN) is a major microvascular complication and the leading cause of end-stage renal disease (ESRD)^1^. Approximately 50% of ESRD patients who need dialysis are diabetic, and they are also highly susceptible to macrovascular complications^2^. However, the underlying molecular mechanisms leading to DN are not fully elucidated, although several classic mechanisms and pathways leading to DN have been described over the years^3^,^4^,^5^,^6^.

MicroRNAs (miRNAs) are endogenously produced short (about 22 nucleotides) noncoding RNAs. These miRNAs are transcribed by RNA polymerase II as long transcripts, called primary miRNAs, and then they are sequentially processed by two RNase III proteins (Drosha and Dicer) in the nucleus and cytoplasm to generate precursor and then mature miRNAs^7^. miRNAs have been shown to play important roles in modulating gene expression and regulating diverse biologic processes^8^,^9^. Moreover, evidence shows that miRNAs regulate the expression of key genes associated with kidney diseases and several miRNAs^10^,^11^,^12^,^13^,^14^,^15^,^16^,^17^. These miRNAs were shown to regulate fibrotic gene expression and glomerular hypertrophy via targeting transforming growth factor (TGF)-β signaling, high glucose (HG) effects and downstream transcription regulators, or extracellular matrix genes. These emerging reports clearly show that several miRNAs are involved in promoting or attenuating the progression of DN by targeting genes related to fibrosis, inflammation, oxidative stress and signal transduction. In addition, some of these miRNAs work in amplifying circuits, while others have autonomous effects and cell-specific roles. It is therefore important to examine the molecular mechanisms underlying the mis-regulated expression of key miRNAs associated with DN. Some reports have shown that miRNAs can be regulated by transcriptional mechanisms, including transcription of their host long non-coding RNA, and the role of chromatin histone acetylation of the miRNA promoter has also been demonstrated^15^,^18^,^19^,^20^,^21^,^22^. However, it is not known whether diabetic conditions can alter miRNA levels by dysregulation of miRNA processing steps.

Methyl-CpG binding protein 2 (MeCP2) is a transcriptional repressor by binding to methylated DNA and recruiting histone deacetylase complex proteins^23^,^24^,^25^,^26^. Interestingly, MeCP2 also regulates gene expression by posttranslational mechanisms involving suppression of nuclear miRNA processing^27^,^28^. They found that phosphorylated MeCP2 (p-MeCP2) binds directly to DiGeorge syndrome critical region 8 (DGCR8), a critical component of the nuclear miRNA-processing machinery^29^,^30^,^31^,^32^ and interferes with the assembly of the Drosha and DGCR8 complex. On the other hand, homeo-domain interacting protein kinase 2 (HIPK2), which is a conserved serine/threonine nuclear kinase and controls gene expression by phosphorylating transcription factors has been shown to phosphorylate MeCP2 at Ser 80, and p-MeCP2 mediated by HIPK2 was suggested to contribute to apoptosis^28^,^33^. HIPK2 plays a role in kidney fibrosis in mice with human immunodeficiency virus (HIV), and HIPK2 expression is higher not only in kidneys of HIV transgenic mice and patients with HIV associated nephropathy, but also in kidneys of patients with focal segmental glomerulosclerosis (FSGS), DN and immunoglobulin A nephropathy (IgA nephropathy)^34^. We previously reported that let-7 miRNA family members are downregulated under diabetic conditions through changes in lin-28b which mediates the processing of let-7^35^. However, it is not known if MeCP2 and HIPK2 are involved in processing and expression of candidate miRNAs that are downregulated in DN.

In this study, we evaluated whether the downregulation of key protective miRNAs, such as miR-25, under diabetic conditions in the kidney are mediated by the mis-regulation of factors mediating the biogenesis and processing of these miRNAs. Specifically, we assessed whether changes in HIPK2 and p-MeCP2 are observed in glomeruli from diabetic mice, and can affect the processing of miR-25 in renal mesangial cells under diabetic conditions.

## Results

### p-MeCP2, HIPK2, and NOX4 expressions are upregulated in glomeruli of diabetic mice

**Figure 1:**
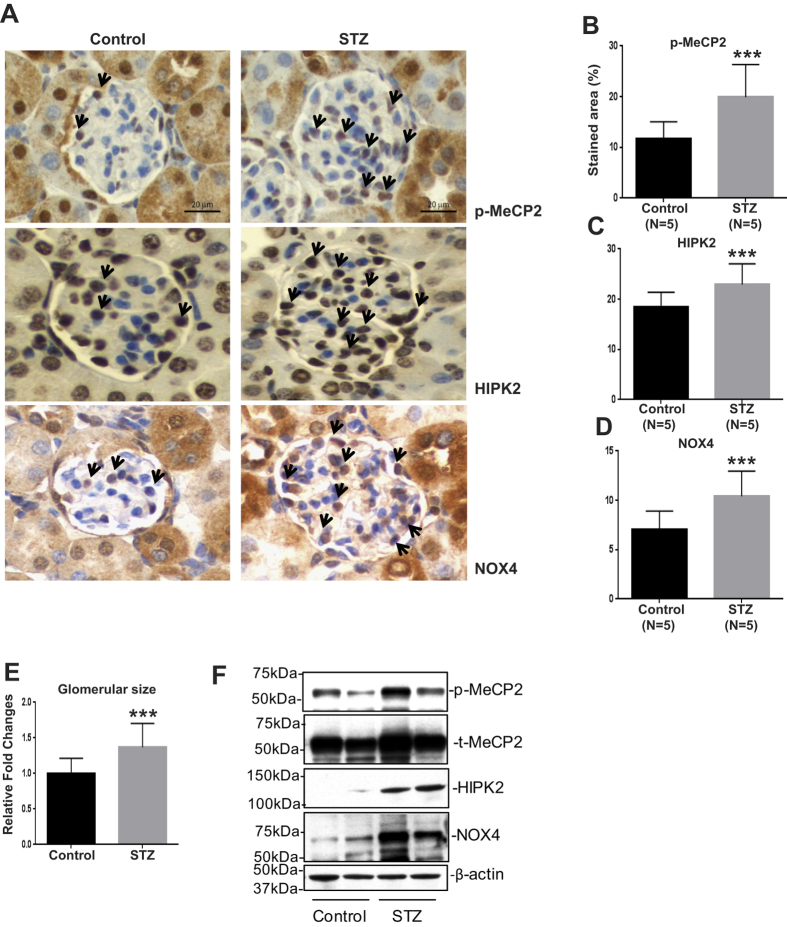
The expression levels of p-MeCP2, HIPK2, and NOX4 are upregulated in glomeruli of diabetic mice. Immunohistochemistry showed that p-MeCP2-, HIPK2-, or NOX4-stained cells (black arrows) were significantly increased in the glomerulus from streptozotocin (STZ) injected-diabetic mice (4 weeks post diabetes injection) compared with those in non-diabetic control mice (**A** to **D**), Representative immunostains of glomerular p-MeCP2, HIPK2, and NOX4). Moreover, the glomerular size was significantly larger in these diabetic mice than that in control mice (**E**). In each group (*n* = 5), more than 30 pictures were used for quantification of immunostaining (Scale bar, 20 um, Mean ± SEM, ***p < 0.001 vs. Control). Western blotting showed that the protein expressions of p- & t-MeCP2, HIPK2, and NOX4 were also higher in the diabetic mice compared to controls (**F**). We used 2 pooled protein lysates collected separately from 2 to 3 sets of mice (non-diabetic control and diabetic mice), respectively. Western blottings were performed 2 to 3 times with these 2 pooled protein lysates and results shown are representative blots. Uncropped scans are presented in Supplementary Fig. 1. Abbreviations; p-MeCP2, phosphorylated methyl-CpG binding protein 2; HIPK2, homeo-domain interacting protein kinase 2; NOX4, NADPH oxidase 4; t-MeCP2, total methyl-CpG binding protein 2.

We first examined whether the expression of p-MeCP2, HIPK2, and NOX4 are altered in the glomeruli of diabetic versus control mice. Samples were obtained four weeks after the onset of diabetes in streptozotocin (STZ) or vehicle injected C57BL6 mice. Immunohistochemistry showed that p-MeCP2-, HIPK2-, or NOX4-stained cells were significantly increased in the glomerulus from diabetic mice compared with control non-diabetic mice (Fig. 1A to D). Glomerular size was significantly larger in these diabetic mice versus control mice indicating increased glomerular hypertrophy (Fig. 1E). Moreover, western blotting also showed that the protein expressions of p- & total (t)-MeCP2, HIPK2, and NOX4 were also higher in the diabetic mice compared to controls (Fig. 1F). These results suggest that glomerular p-MeCP2, HIPK2, and NOX4 are related to the pathology of DN.

### *Nox4* mRNA expression is increased, but *Siah1* mRNA expression is decreased in diabetic conditions

**Figure 2:**
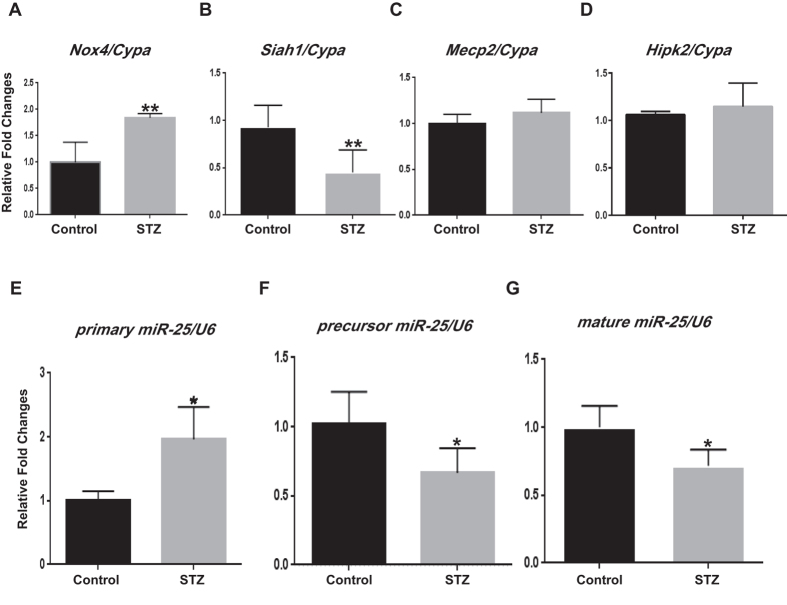
*Nox4* mRNA and primary miR-25 expression levels are upregulated, while *Siah1* expression as well as precursor and mature miR-25 expression levels are downregulated in glomeruli from diabetic mice. The mRNA expression of *Nox4* was significantly upregulated in the glomeruli of the diabetic mice compared with non-diabetic control mice (**A**), while *Siah1* mRNA expression was significantly downregulated in diabetes compared to control (**B**). However, there were no significant differences in the mRNA expressions of *Mecp2* and *Hipk2* between the two groups (**C** and **D**). The expression of primary miR-25 was significantly increased in the glomeruli of diabetic mice compared with that in control mice (**E**), whereas precursor and mature miR-25 expressions were significantly decreased in the diabetic mice compared to control (**F** and **G**). We used RNA extracted from five glomeruli in each of the non-diabetic control and diabetic group, respectively. qPCR experiments for RNA expression were conducted 3 to 4 times with these respective 5 separate glomerular samples in each experiment (Mean ± SEM, *p < 0.05, and **p < 0.01 vs. Control). PCR data shown is normalized to *cyclophilin A (Cypa)* or U6 RNA Abbreviations; STZ, streptozotocin; NOX4, NADPH oxidase 4; SIAH1, seven in absentia homolog 1; MeCP2, methyl-CpG binding protein 2; HIPK2, homeo-domain interacting protein kinase 2.

We found that mRNA levels of *Nox4* were significantly increased in the glomeruli of the diabetic mice compared to control, but Seven in Absentia Homolog 1 (*Siah1*) mRNA expression was significantly decreased in diabetes compared to control (Fig. 2A and B). However, there were no significant differences in the mRNA expressions of *Mecp2* and *Hipk2* between the two groups (Fig. 2C and D), suggesting that the expression of these proteins may be regulated by post-translational mechanisms.

### Primary miR-25 expression is increased, while precursor and mature miR-25 expressions are decreased in diabetic conditions

The levels of several miRNAs have been reported to be decreased in DN^15^,^35^,^36^,^37^,^38^,^39^,^40^,^41^,^42^,^43^,^44^,^45^. Among these miRNAs and also decreased miRNAs identified by small RNA sequencing in glomeruli from STZ diabetic mice^22^, we confirmed that miR-25 and miR-93 expressions were significantly downregulated in TGF-β treated mouse mesangial cells (MMCs), but the expression of only miR-25 was consistently and significantly decreased even in HG-treated MMCs relative to control. Since we previously showed that let-7 family (lin28-mediated)^35^ and miR-130b^44^ were downregulated under diabetic conditions by mechanisms not involving p-MeCP2, we focused on miR-25 in this study. The expression of primary miR-25 was significantly upregulated in the glomeruli of diabetic mice compared with that in the control (Fig. 2E), whereas precursor and mature miR-25 levels were significantly downregulated in the diabetic mice compared to control (Fig. 2F and G).

### p-MeCP2, HIPK2, and NOX4 expressions are upregulated, but SIAH1 expression is downregulated in HG and TGF-β treated MMCs

**Figure 3:**
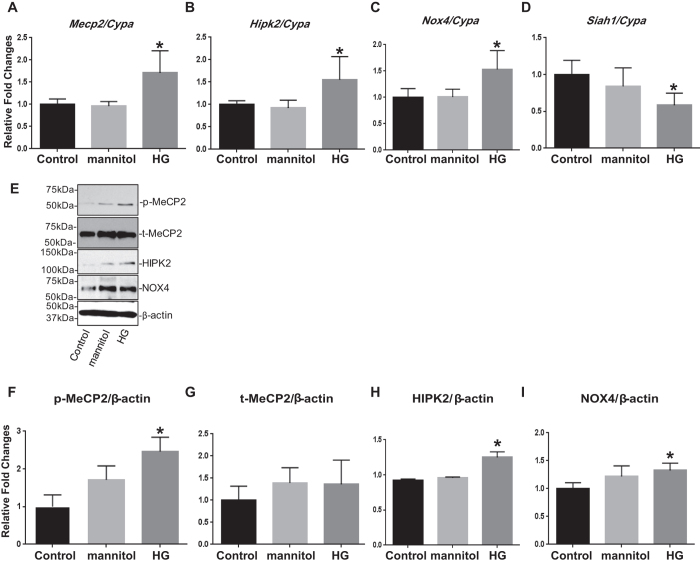
p-MeCP2, HIPK2, and NOX4 expressions are upregulated, but SIAH1 mRNA expression is downregulated in HG treated MMCs. The mRNA expressions of *Mecp2, Hipk2*, and *Nox4* (**A** to **C**), as well as protein expressions of p-MeCP2, HIPK2, and NOX4 (**E,F,H** and **I**) were significantly upregulated in the HG (72 hrs) treated MMCs compared to those in the control group (NG), while mRNA expression of *Siah1* was significantly decreased under these conditions (**D**). There was no significant difference in the protein expression of t-MeCP2 among the groups (**E** and **G**). qPCRs were performed 3 to 4 times with RNA isolated from 3 independent cell culture experiments. Western blotting was also performed 2 to 3 times with protein lysates derived from 3 independent cell culture experiments, and representative blots are shown (Mean ± SEM *p < 0.05 vs. NG). Uncropped scans are presented in Supplementary Fig. 1. Abbreviations; NG, normal glucose; HG, high glucose; MeCP2, methyl-CpG binding protein 2; HIPK2, homeo-domain interacting protein kinase 2; NOX4, NADPH oxidase 4; SIAH1, seven in absentia homolog 1; p-MeCP2, phosphorylated methyl-CpG binding protein 2; t-MeCP2, total methyl-CpG binding protein 2.

To further clarify whether the glomerular expressions of p-MeCP2, HIPK2, and NOX4 are regulated by diabetic conditions *in vitro*, MMCs were cultured in either normal glucose (NG, 5.5 mM) or high glucose (HG, 25 mM) or equimolar osmotic control mannitol for 72 hrs. The mRNA levels of *Mecp2* and *Hipk2* (Fig. 3A to C) and protein expressions of p-MeCP2, HIPK2, and NOX4 (Fig. 3E,F,H and I) were significantly upregulated in the HG treated MMCs compared to those in the control group, while mRNA expression of *Siah1* was significantly decreased in the HG treated MMCs compared with controls (Fig. 3D). Protein levels of t-MeCP2 were not significantly different between the three groups (Fig. 3E and G).

**Figure 4:**
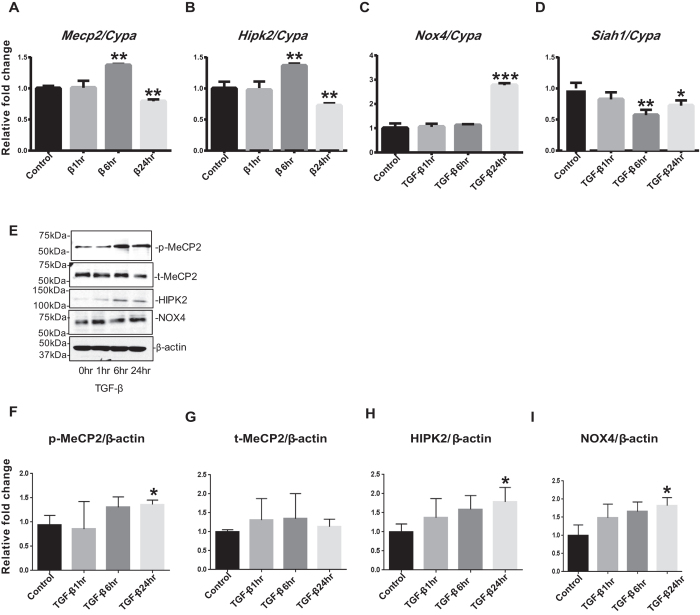
p-MeCP2, HIPK2, and NOX4 expressions are also increased, and SIAH1 mRNA expression is decreased in TGF-β treated MMCs. The mRNA expressions of *Mecp2* and *Hipk2* were significantly upregulated in the 6 hr TGF-β treated MMCs compared with those in the control group, while these changes returned below control in the 24 hr TGF-β treated MMCs relative to control (**A** and **B**). *Nox4* mRNA levels were significantly increased in the 24 hr TGF-β treated MMCs compared to control (**C**), whereas mRNA expressions of *Siah1* were significantly decreased in 6 & 24 hr TGF-β treated MMCs compared to control (**D**). The protein expressions (**E, F, H,** and **I**) of p-MeCP2, HIPK2, and NOX4 were revealed to be significantly increased in the 24 hr TGF-β treated MMCs relative to control, whereas there was no significant difference in t-MeCP2 protein expression among the groups (**E** and **G**). qPCRs were performed 3 to 5 times with RNA isolated from 3 independent cell culture experiments. Western blotting was also performed 3 to 4 times with protein lysates derived from 3 independent cell culture experiments, and representative blots are shown (Mean ± SEM *p < 0.05, **p < 0.01, and ***p < 0.001 vs. Control). Uncropped scans are presented in Supplementary Fig. 2. Abbreviations; MeCP2, methyl-CpG binding protein 2; HIPK2, homeo-domain interacting protein kinase 2; NOX4, NADPH oxidase 4; SIAH1, seven in absentia homolog 1; p-MeCP2, phosphorylated methyl-CpG binding protein 2; t-MeCP2, total methyl-CpG binding protein 2.

We found that these factors were also similarly regulated by TGF-β. Thus, in TGF-β (10 ng/ml) treated MMCs, the mRNA levels of *Mecp2* and *Hipk2* were significantly increased by 6 hr after treatment, and returned below normal by 24 hr compared with the control group (Fig. 4A and B). mRNA expression of *Nox4* was significantly upregulated in the 24 hr TGF-β treated MMCs compared to control (Fig. 4C). On the other hand, *Siah1* mRNA levels were significantly decreased in the 6 & 24 hr TGF-β treated MMCs compared to control (Fig. 4D). Western blots showed that the protein levels (Fig. 4E,F,H and I) of p-MeCP2, HIPK2, and NOX4 were also significantly increased in the 24 hr TGF-β treated MMCs relative to those in the control group, whereas there was no significant difference in t-MeCP2 protein expression among the groups (Fig. 4G). Taken together, p-MeCP2, HIPK2, and NOX4 expressions are increased, but mRNA expression of *Siah1* is decreased in mesangial cells under diabetic conditions *in vivo* and *in vitro*.

### Primary miR-25 expression is increased, while precursor and mature miR-25 expressions are decreased in HG and TGF-β treated MMCs

**Figure 5:**
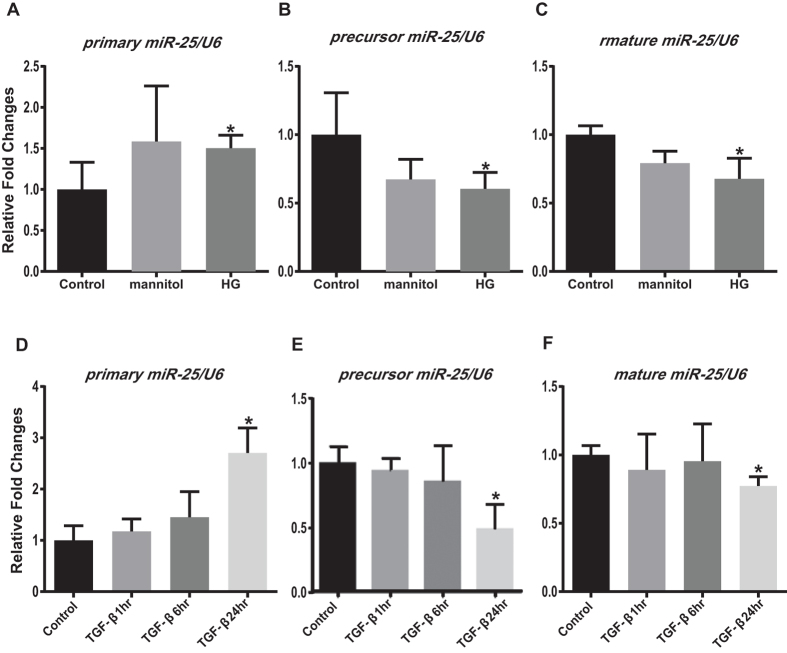
Primary miR-25 expression is upregulated, while precursor and mature miR-25 expressions are downregulated in HG and TGF-β treated MMCs. Levels of primary miR-25 were significantly increased in the HG and 24 hr TGF-β treated MMCs compared to respective controls (**A** and **D**), while the expressions of precursor and mature miR-25 were significantly decreased in the treated group compared to the respective controls (**B** and **C, E** and **F**). qPCRs were performed 3 to 4 times with RNA isolated from 3 independent cell culture experiments (Mean ± SEM *p < 0.05 vs. NG or Control). Abbreviations; NG, normal glucose; HG, high glucose.

The expression levels of primary miR-25 were significantly increased in the HG and 24 hr TGF-β treated MMCs compared with those in the control, while the expressions of precursor and mature miR-25 were significantly decreased in the treated group compared to the control (Fig. 5A to F). These results suggest that initial step of miRNA processing may be inhibited by diabetic conditions because, although primary miR-25 expression is upregulated, precursor and mature miR-25 expressions are downregulated in mesangial cells under diabetic milieu *in vivo* and *in vitro*.

### HG and TGF-β induced increases in mRNA and protein expressions of p-MeCP2 and NOX4 in MMCs are attenuated by knockdown of HIPK2

**Figure 6:**
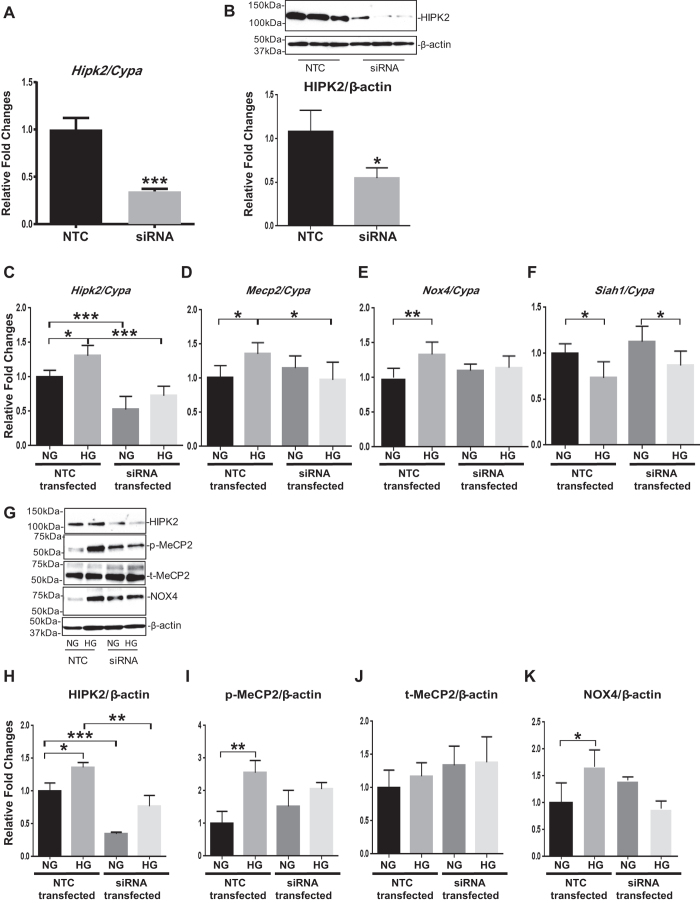
HG induced increases in mRNA and protein expressions of p-MeCP2 and NOX4 are attenuated by knockdown of *Hipk2*. The expressions of *Hipk2* mRNA (**A**) as well as HIPK2 protein levels (**B**) were significantly decreased after *Hipk2* gene silencing by transfection with specific siRNAs (15 nM of *Hipk2* siRNA) relative to nontargeting control siRNA (NTC) (Mean ± SEM *p < 0.05, and ***p < 0.001 vs. NTC). Significant reductions of HIPK2 mRNA and protein (basal and HG induced) by *Hipk2* siRNA in MMC were confirmed (**C** and **H**). Moreover, in parallel, the mRNA levels of *Mecp2* and *Nox4* (**D** and **E**) and protein expressions of p-MeCP2 and NOX4 (**G,I** and **K**) that were significantly increased after HG treatment in NTC transfected MMCs, and attenuated in *Hipk2* siRNA transfected MMCs. However, the decrease in *Siah1* mRNA expression after HG treatment was not affected even after knockdown of *Hipk2* (**F**). qPCRs were performed 3 to 5 times with RNA isolated from 3 independent cell culture experiments. Western blotting was also performed 3 to 4 times with protein lysates derived from 3 independent cell culture experiments, and representative blots are shown (Mean ± SEM *p < 0.05, **p < 0.01, and ***p < 0.001 vs. NG). Uncropped scans are presented in Supplementary Figs 2 and 3. Abbreviations; NTC, nontargeting control siRNA; HIPK2, homeo-domain interacting protein kinase 2; NG, normal glucose; HG, high glucose; MeCP2, methyl-CpG binding protein 2; NOX4, NADPH oxidase 4; SIAH1, seven in absentia homolog 1; p-MeCP2, phosphorylated methyl-CpG binding protein 2; t-MeCP2, total methyl-CpG binding protein 2.

Next, we investigated the effect of siRNA mediated knockdown of *Hipk2* on MeCP2 phosphorylation, miR-25 processing, and NOX4 expression. *Hipk2* gene silencing by specific siRNAs (15 nM of *Hipk2* siRNA) resulted in a significant decrease in *Hipk2* mRNA (Fig. 6A) as well as HIPK2 protein levels (Fig. 6B) relative to nontargeting control siRNA (NTC). Significant reductions of *Hipk2* mRNA and protein (basal and HG induced) by *Hipk2* siRNA in MMC were confirmed (Fig. 6C,G and H). Moreover, the increases in mRNA (Fig. 6D and E) expressions of MeCP2 and NOX4 and protein (Fig. 6G,I and K) expressions of p-MeCP2 and NOX4 observed after HG treatment in NTC transfected MMCs were attenuated in *Hipk2* siRNA transfected MMCs. However, the downregulation of *Siah1* mRNA expression observed in HG treated cells was not affected by the knockdown of *Hipk2* (Fig. 6F). Taken together, the downregulated HIPK2 (by *Hipk2* siRNA) in HG treated mesangial cells has functional outcomes to downregulate MeCP2 phosphorylation and NOX4 expression, while siHIPK2 had no effect on SIAH1, confirming that SIAH1 is upstream of HIPK2.

**Figure 7:**
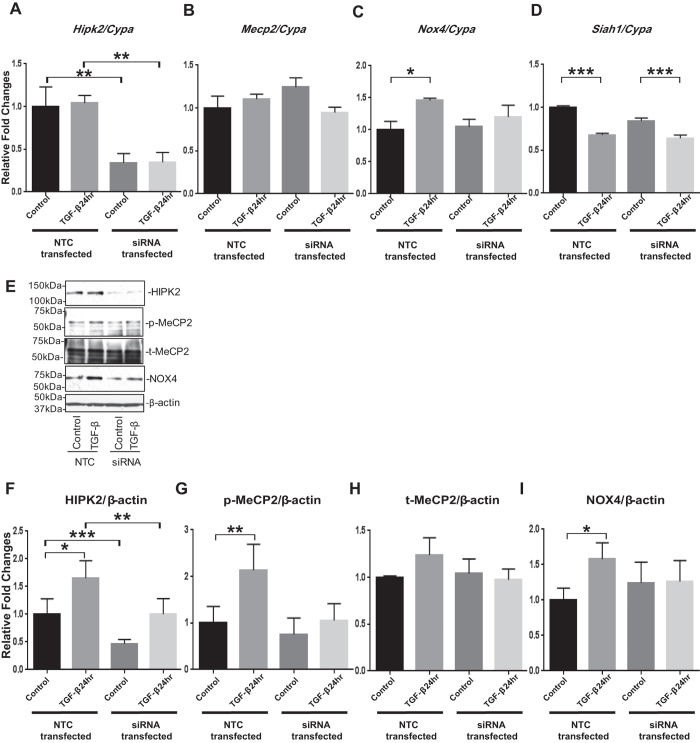
TGF-β-induced increases in mRNA and protein expressions of p-MeCP2 and NOX4 are attenuated by knockdown of *Hipk2* in MMCs. Significant reductions of HIPK2 mRNA and protein by *Hipk2* siRNA in MMC (basal and TGF-β treated) were confirmed (**A** and **F**). The increases in mRNA expression of NOX4 seen with 24 hr TGF-β treatment in the NTC transfected MMCs were also attenuated by knockdown of *Hipk2* in MMC (**C**). However, there were no significant differences in mRNA expressions of *Hipk2* and *Mecp2* between control and TGF-β treated MMCs under these conditions (**A** and **B**). Moreover, the protein expressions of p-MeCP2 and NOX4 that were significantly upregulated in the NTC transfected MMCs with 24 hr TGF-β treatment were also attenuated by knockdown of *Hipk2* in MMCs (**E,G,** and **I**). However, *Siah1* mRNA expression level was still significantly decreased after TGF-β treatment even after knockdown of *Hipk2* (**D**). qPCRs were performed 3 to 5 times with RNA isolated from 3 independent cell culture experiments. Western blotting was also performed 3 to 5 times with protein lysates derived from 3 independent cell culture experiments, and representative blots are shown (Mean ± SEM *p < 0.05, **p < 0.01, and ***p < 0.001 vs. Control). Uncropped scans are presented in Supplementary Fig. 3. Abbreviations; NTC, nontargeting control siRNA; HIPK2, homeo-domain interacting protein kinase 2; MeCP2, methyl-CpG binding protein 2; NOX4, NADPH oxidase 4; SIAH1, seven in absentia homolog 1; p-MeCP2, phosphorylated methyl-CpG binding protein 2; t-MeCP2, total methyl-CpG binding protein 2.

We next examined the effects of *Hipk2* siRNA on TGF-β actions. Significant reductions of *Hipk2* mRNA and protein (basal and 24hr TGF-β induced) by *Hipk2* siRNA in MMC were confirmed (Fig. 7A,E and F), whereas MeCP2 showed no change (Fig. 7B,E and H). The increase in *Nox4* mRNA expression (Fig. 7C) in cells treated with TGF-β for 24 hr that was evident in the NTC transfected MMCs, was significantly attenuated in the MMCs transfected with *Hipk2* siRNA. Moreover, the significant increases in protein expressions of p-MeCP2 and NOX4 that were evident in NTC transfected MMCs treated for 24 hr with TGF-β were also attenuated in the MMCs by knockdown of *Hipk2* (Fig. 7E,G and I). However, the decrease in *Siah1* mRNA after TGF-β treatment (Fig. 7D) was not affected by the siRNA mediated knockdown of *Hipk2*. Taken together, these results demonstrate that HIPK2, which is upregulated in HG and TGF-β treated mesangial cells, is also involved in MeCP2 phosphorylation and the expression of NOX4, whereas, because *Hipk2* siRNA had no effect on SIAH1, it again confirms that SIAH1 is upstream of HIPK2.

### Increased primary miR-25 expression, but decreased precursor and mature miR-25 expression levels are also attenuated by knockdown of HIPK2 even in HG and TGF-β treated MMCs

**Figure 8:**
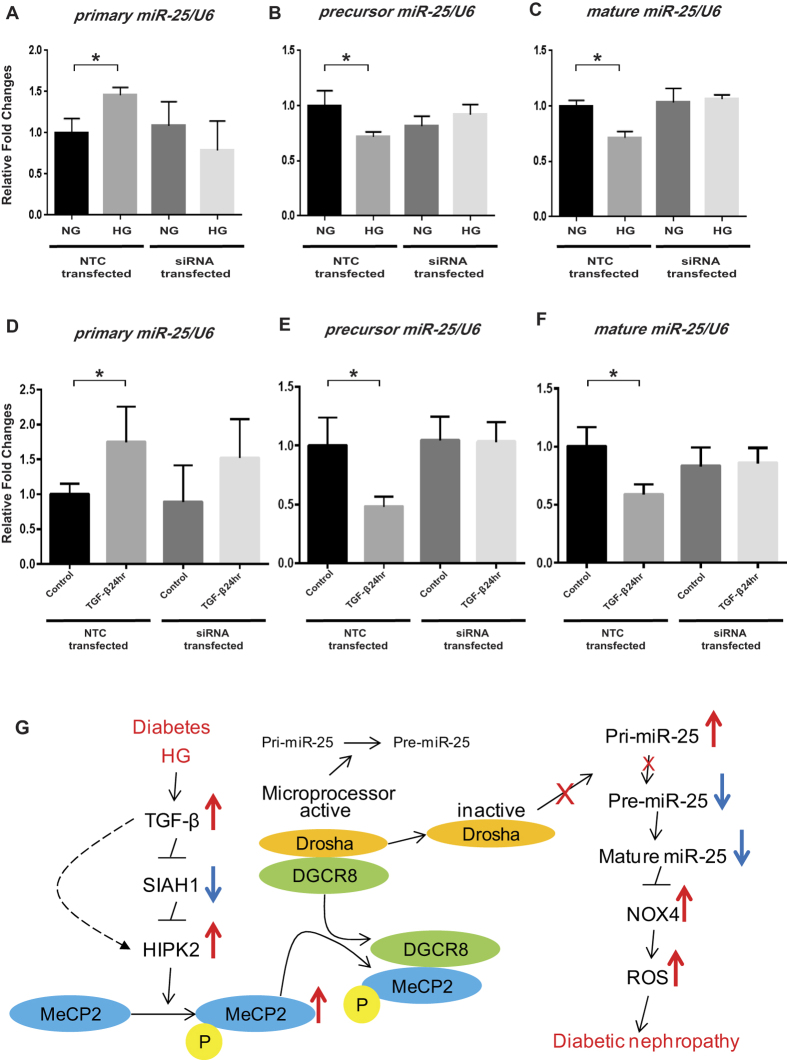
Increased primary miR-25 expression, but decreased precursor and mature miR-25 expression levels are attenuated by knockdown of *Hipk2* in HG and TGF-β treated MMCs. The expression of primary miR-25 was significantly increased, but expressions of precursor and mature miR-25 were significantly decreased after HG (**A** to **C**) and 24 hr TGF-β treatment (**D** to **F**) in NTC transfected MMCs. However, these increases were also attenuated in *Hipk2* siRNA transfected MMCs relative to NTC transfected MMCs. qPCRs were performed 3 to 5 times with RNA isolated from 3 independent cell culture experiments (Mean ± SEM *p < 0.05 vs. NG or Control). Abbreviations; NTC, nontargeting control siRNA; NG, normal glucose; HG, high glucose. (**G**) MeCP2 regulated by HIPK2 stabilized by decreased SIAH1 under diabetic conditions *in vitro* (HG and TGF-β) or *in vivo* plays a key role in suppressing miR-25 processing and expression. Consequently, NOX4, a target of miR-25 and an inducer of oxidative stress, can be upregulated due to downregulation of miR-25. This illustrates a mechanism for the regulation of miRNAs associated with the pathogenesis of DN. Abbreviations; DGCR8, DiGeorge syndrome critical region 8; pri-miR, primary microRNA; pre-miR, precursor microRNA; NOX4, NADPH oxidase 4; ROS, reactive oxygen species; P-MeCP2, phosphorylated MeCP2.

The primary miR-25 was significantly upregulated, but expressions of precursor and mature miR-25 were significantly decreased after HG and 24 hr TGF-β treatment in NTC transfected MMCs. However, these changes were also attenuated in *Hipk2* siRNA transfected MMCs relative to NTC (Fig. 8A to F). These results suggest that MeCP2 phosphorylated by HIPK2 stabilized under diabetic conditions can block the first step of miR-25 processing and thus reduce the levels of precursor and mature miR-25.

## Discussion

In this study, we showed for the first time that miR-25 expression can be downregulated under diabetic conditions due to the inhibition of the first step of miR-25 processing by p-MeCP2. The levels of p-MeCP2 were enhanced by the kinase HIPK2, whose expression is augmented due to stabilization by the downregulation of SIAH1 under diabetic conditions *in vitro* and *in vivo*. We also observed that NOX4, a validated target of miR-25 and a known inducer of oxidative stress^37^,^46^,^47^, was increased in MMC treated with HG and TGF-β (diabetic conditions) and in the glomeruli of diabetic mice (*in vivo*) relative to corresponding controls, suggesting that downregulation of miR-25 via inhibition of its processing may be a new mechanism by which NOX4 and ensuing oxidant stress in augmented in the pathogenesis of DN. Together with the well-known role of NOX4 in the pathogenesis of DN, including data showing that genetic or pharmacological inhibition of *Nox4* attenuated parameters of DN in mouse models^48^,^49^,^50^, our results further support the significance of this SIAH1/HIPK2/MeCP2/miR-25/NOX4 pathway in DN. Besides NOX4, several other targets of miR-25 have been reported^51^,^52^,^53^,^54^, which might also contribute to signaling pathways in DN. However, as supported by several reports^46^,^47^,^48^,^49^,^50^. NOX4 is one of the most relevant targets of miR-25 that is related to DN pathology. Glomerular mesangial hypertrophy and ECM accumulation induced by HG, TGF-β, oxidant stress and related stimuli are relatively early events in the pathogegesis of DN, which can trigger pathological effects in other renal cells (podocytes, tubular, endothelial cells) and ultimately lead to end stage of renal disease. Inhibition of such early features of DN can prevent disease progression, proteinuria and renal failure. Therefore, increased understanding of the mechanisms mediating the early stages of DN is important^12^. Alterations in processing of miRNAs such as miR-25 by p-MeCP2 could be one such mechanism.

Recently miRNAs have been widely studied as novel mechanistic regulators of DN progression. Several miRNAs are dysregulated in early DN and can promote the expression of extracellular matrix proteins and other genes associated with the initial stages of DN^12^. Moreover, several studies using renal cells *in vitro*, and *in vivo* animal models have shown functional relationships between aberrant expression of miRNAs and genes as well as pathways related to renal fibrosis and DN^12^,^13^,^18^,^20^,^22^,^39^,^40^,^45^,^55^,^56^,^57^,^58^. However, it is not still clear whether changes in miRNA processing mechanisms are associated with the observed changes in the expression of key miRNAs implicated in DN. We hypothesized that MeCP2 phosphorylated by HIPK2 (p-MeCP2) may affect miRNA processing in diabetic kidney disease based on recent reports showing the role of p-MeCP2 in suppressing nuclear miRNA processing^27^ and that HIPK2 can phosphorylate MeCP2 at Ser 80^33^. Moreover, evidence shows that HIPK2 is stabilized and activated after disruption of HIPK2-SIAH1 complex in response to DNA damage^59^,^60^. Our results revealed associations between SIAH1, HIPK2 and p-MeCP2 levels and processing of miRNAs, such as miR-25 which are downregulated by factors related to DN. Although loss-of-function mutations in the *MeCP2* gene itself are found in the Rett syndrome^61^,^62^, the pathogenesis of DN is different, since p-MeCP2 regulates miRNA processing contributing to the expression of detrimental factors like NOX4.

As indicated above, p-MeCP2, HIPK2, and NOX4 were expressed at higher levels under diabetic conditions (*in vivo* and *in vitro*) compared with normal conditions, whereas the mRNA expression of *Siah1* was decreased. Moreover, the increases in p-MeCP2 and NOX4 expressions after HG and 24 hr TGF-β treatments were attenuated in MMC transfected with siRNAs targeting *Hipk2*, whereas the decreases in *Siah1* mRNA levels under these conditions were not altered by these treatments. These results suggest that SIAH1 suppressed by HG and/or TGF-β can stabilize HIPK2, and the subsequent increases in HIPK2 (via stabilization) can augment the phosphorylation of MeCP2 via the kinase activity of HIPK2. We also observed that the expression levels of mature and precursor miR-25 were decreased, but primary miR-25 levels were increased *in vivo* in the diabetic mice. Similar trends were also seen in MMC treated with HG or TGF-β (24 hr), and moreover these changes were attenuated by siRNAs targeting *Hipk2* relative to NTC. These data further substantiate the role of HIPK2 as a critical kinase of MeCP2 as reported^33^ and that increased p-MeCP2 mediated by HIPK2 stabilized under the diabetic conditions can block miR-25 processing from the primary to precursor conversion steps. Importantly, since NOX4 is a direct target of miR-25^37^, the resultant decreases in miR-25 can lead to enhance NOX4 expression (and related oxidative stress) as seen in diabetes. Conversely, knockdown of *Hipk2* can prevent the induction of NOX4 by restoring miR-25 processing and mature miR-25 levels. Based on these findings, a schematic model for miR-25 processing and NOX4 expression in the early stage of DN is depicted in Fig. 8G. Our studies do not fully address the functional *in vivo* role of this pathway from SIAH1 to miR-25 and NOX4 via HIPK2 and p-MeCP2. This could be assessed in the future by treating diabetic mice with *Hipk2* siRNAs.

As suggested earlier that MeCP2 and HIPK2 protein expressions may be regulated at the posttranslational level^63^, we also observed that the mRNA expressions of *Mecp2* and *Hipk2* were not significantly different in the glomeruli of diabetic versus control mice (Fig. 2C and D), although the protein levels of p-MeCP2 and HIPK2 were significantly upregulated *in vivo* in the glomeruli of diabetic versus control mice (Fig. 1F). Interestingly however, mRNA expressions of *Mecp2* and *Hipk2* were significantly increased *in vitro* in MMCs treated only for 6 hr TGF-β, but this increase was lost and even reduced below control after 24 hr of TGF-β treatment compared to control (Fig. 4A and B). Expressions of these two factors were also significantly upregulated in MMCs treated with HG relative to control (Fig. 3A and B). These results suggest that exogenous short-term treatment with stimuli like TGF-β (e.g. 6 hr) may upregulate mRNA expression of *Mecp2* and *Hipk2* by transcriptional regulation, possibly via Smads, and that these effects may be lost at later time points (24 hr treatment with exogenous TGF-β). In cells treated with HG for 72 hr, endogenous TGF-β can be increased^18^ which can account for the increases in *Hipk2* mRNA levels. However, *in vivo* (4 week-STZ injected diabetic mice), the duration of hyperglycemia is relatively longer and hence upregulation of endogenous TGF-β is also longer, suggesting that HIPK2 protein expression under these *in vivo* conditions is likely to be upregulated at the posttranslational level (stabilization through decreases in SIAH1). Taken together, these data show that TGF-β can modulate HIPK2 expression by two independent mechanisms. Since we found three repeats of consensus Smad binding sites (CAGA) 3 kb upstream of the *Hipk2* promoter, one mechanism for *Hipk2* mRNA upregulation by TGF-β may be enhanced transcription via rapid Smad activation as reported previously^18^,^22^,^35^ and binding to the *Hipk2* promoter. In the second mechanism, HIPK2 protein can be stabilized and enhanced posttranslationally via decrease of SIAH1 as seen under diabetic conditions *in vivo* (Fig. 8G)^34^,^59^,^60^,^63^.

Notably, the mechanism described in this study is likely also relevant to human DN and other human kidney diseases, because MeCP2 expression has been reported to be increased in the kidneys of patients with chronic kidney disease, lupus nephritis, FSGS, IgA nephropathy as well as DN (https://www.nephroseq.org/resource/main.html). In summary, these data demonstrate that MeCP2 regulated by HIPK2 stabilized by decreases in SIAH1 under diabetic conditions plays an important role in suppressing miR-25 processing and expression. As a consequence, NOX4, a target of miR-25, can be upregulated and this leads to oxidant stress associated with the pathology of DN (Fig. 8G). Together, these results reveal a novel mechanism for downregulation of key protective miRNAs in the diabetic kidney and also new therapeutic targets for the prevention of DN.

## Methods

### Animals

All animal studies were conducted according to a protocol approved by the Institutional Animal Care and Use Committee at the Beckman Research Institute of City of Hope. C57BL/6 mice (The Jackson Laboratory) were injected with 50 mg/kg of streptozotocin (STZ) intraperitoneally on 5 consecutive days. Mice injected with diluent served as controls. Diabetes was confirmed by tail vein blood glucose levels (fasting glucose >300 mg/dl). Each group was composed of five mice. All mice were sacrificed at 4 weeks post-induction of diabetes. Glomeruli were isolated from freshly harvested kidneys by a sieving technique^58^. Enriched glomerular tissue below the sieve was collected and transferred to another sieve with a pore size of 75 μm. After several washes with cold PBS, the glomerular tissue remaining on top of the sieve was collected. The glomeruli were collected for protein and RNA extraction. RNA samples were isolated from glomeruli of individual mice. However, for Western blots, because protein amounts obtained from glomeruli of single mice are not sufficient, we pooled glomeruli from two or three mice for protein extraction.

### Cell Culture Experiments

MMCs were obtained and cultured as described previously in RPMI 1640 medium supplemented with 10% FBS^55^. Passages 6–8 were used for experiments. Recombinant human TGF-β1 was from R&D Systems (Minneapolis, MN).

### Immunohistochemistry

Formalin-fixed, paraffin-embedded sections of mouse kidneys were mounted onto positively charged slides, deparaffinized, washed with water, blocked with Dako protein block (Dako, Carpinteria, CA), and incubated with p-MeCP2 antibody (1:100, #MP4601 from ECM Biosciences), HIPK2 antibody (1:100, #ab28507 from abcam), and NOX4 antibody (1:100, #ab133303 from abcam) for 30 min, respectively. Slides were washed with Dako wash, treated with hydrogen peroxide for 5 min, washed with PBS, incubated with anti-rabbit secondary antibody conjugated with a peroxidase polymer (Dako, Carpinteria, CA), and washed and incubated with 3,3′-diaminobenzidine for 8 min. Slides were counterstained with hematoxylin and mounted. Images were taken at x40 magnification using an Olympus BX51 microscope with In Studio (Pixera Corp., Santa Clara, CA) software to collect images. ImagePro software (Media Cybernetics Inc., Rockville, MD) was used to quantify staining.

### Real Time Quantitative PCR

RNA was extracted using miRNeasy columns (Qiagen, Inc. Valencia, CA). The analyses of miRNA expressions (primary, precursor, and mature) were performed with the qScript miRNA cDNA synthesis kit (Quanta Biosciences, Gaithersburg, MD) and PerfeCTa SYBR Green Supermix (Quanta Biosciences). GeneAmp RNA PCR kit (Applied Biosystems, Carlsbad, CA) and POWER SYBR Green mix (Applied Biosystems) were used for mRNA quantification. Primary and precursor miRNAs were quantified with quantitative real-time PCR assays using specific primary and precursor primers from Qiagen, and mature miRNA was amplified with specific mature miRNA sequences as forward primers and the universal primer provided in the kit as reverse primer. Real time quantitative PCRs were performed on the 7500 real time PCR system (Applied Biosystems, Foster City, CA). The expression of regular mRNA was normalized to *cyclophilin A (Cypa)* and small RNAs were normalized to U6 RNA, as previously described^18^,^20^,^22^,^35^,^44^,^58^.

PCR primer sequences were as follows:

*primary miR-25* forward, 5′-CTCCCTCACAGGACAGCTGAACAC-3′

*primary miR-25* reverse, 5′-CTGCCCCCCCACATCTGCAGT-3′

*Precursor miR-25* forward, 5′-GGAGACTTGGGCAATTGCTG -3′

*Precursor miR-25* reverse, 5′-ACCGAGACAAGTGCAATGCC-3′

*Mature miR-25,* 5′-CATTGCACTTGTCTCGGTCTGA-3′

*Hipk2* forward, 5′-GCCGAGAGCGGAGACACA-3′

*Hipk2* reverse, 5′-CTCAGCCTCAGTGGGAATCTG-3′

*Mecp2* forward, 5′-CATACATAGGTCCCCGGTCA-3′

*Mecp2* reverse, 5′-CAGGCAAAGCAGAAACATCA-3′

*Siah1* forward, 5′-AAGTGTCCACCATCCCAGAG-3′

*Siah1* reverse, 5′-ATGTAAGTTTGGGGCGACAG-3′

*Nox4* forward, 5′-TGTTGGGCCTAGGATTGTGTT-3′

*Nox4* reverse, 5′-AGGGACCTTCTGTGATCCTCG-3′

### Western Blot Analysis

Immunoblotting was performed as described previously^55^. Cells were lysed in Laemmli’s sample buffer. Lysates were fractionated on 10% SDS-polyacrylamide gels (Bio-Rad) and transferred to nitrocellulose membrane. Membranes were immunoblotted with appropriate antibodies. Antibodies against p-MeCP2 (1:1,000, #MP4601 from ECM Biosciences), t-MeCP2 (1:1,000, #3456 from Cell Signaling), HIPK2 (1:1,000, #ab28507 from abcam), and NOX4 (1:2,000, #ab133303 from abcam) were used. Antibody against β-actin from Cell Signaling was also used. Blots were scanned using GS-900 densitometer and quantified with Quantity One software (Bio-Rad).

### Transfection of MMCs

Cells (1 × 10^6^/transfection) were transfected with siRNA using an Amaxa Nucleofector (Lonza, Basel, Switzerland) according to the manufacturer’s protocols as described previously^20^. MMCs were trypsinized and resuspended in Basic Nucleofection Solution at 1 × 10^7^/ml. Subsequently, 100 μl of cell suspension (1 × 10^6^ cells) was mixed with 15 nM of ON-TARGET plus siRNA or negative controls (Thermo Fischer Scientific Inc., Waltham, MA) as indicated. Transfected cells were harvested for RNA and protein isolation at indicated times.

### Statistical Analysis

Statistical analysis was performed using PRISM software (Graph-Pad, San Diego, CA) for data analysis with unpaired Student *t* tests for two groups or ANOVA with Dunnett’s post tests for multiple groups. *p* < 0.05 was considered statistically significant. All data were expressed as means ± S.E.

## Additional Information

**How to cite this article**: Oh, H. J. *et al*. Inhibition of the processing of miR-25 by HIPK2-Phosphorylated-MeCP2 induces NOX4 in early diabetic nephropathy. *Sci. Rep.* **6**, 38789; doi: 10.1038/srep38789 (2016).

**Publisher's note:** Springer Nature remains neutral with regard to jurisdictional claims in published maps and institutional affiliations.

## Supplementary Material

Supplementary Information
