# Erratum: Inhibition of the processing of miR-25 by HIPK2-Phosphorylated-MeCP2 induces NOX4 in early diabetic nephropathy

**DOI:** 10.1038/srep40832

**Published:** 2017-01-23

**Authors:** Hyung Jung Oh, Mitsuo Kato, Supriya Deshpande, Erli Zhang, Sadhan Das, Linda Lanting, Mei Wang, Rama Natarajan

Scientific Reports 6: Article number: 3878910.1038/srep38789; published online: 12 12 2016; updated: 01 23 2017

The original version of this Article contained an error in the spelling of the author Sadhan Das which was incorrectly given as Das Sadhan. These errors have now been corrected in the PDF and HTML versions of the Article.

