# Supplementary material for: Inhibition of the processing of miR-25 by HIPK2-Phosphorylated-MeCP2 induces NOX4 in early diabetic nephropathy
Source: Sci Rep. 2016 Dec 12;6:38789. doi: 10.1038/srep38789 (PMC5150532; doi:10.1038/srep38789)
Supplement: Supplementary Information [file srep38789-s1.pdf]

Supplementary Infomation

Inhibition of the processing of miR-25 by HIPK2-Phosphorylated-MeCP2 induces NOX4 in early diabetic nephropathy

Hyung Jung Oh[1,2], Mitsuo Kato[1], Supriya Deshpande[1], Erli Zhang[1,3], Sadhan Das[1], Linda Lanting[1], Mei Wang[1], and Rama Natarajan[1]

[1]*Department of Diabetes Complications and Metabolism, Beckman Research Institute of City of Hope, Duarte, California;* [2]*Ewha Institute of Convergence Medicine, Ewha Womans University Mokdong Hospital, South Korea;* [3]*Tsingua University, Beijing, China*

Correspondence to:

Mitsuo Kato, mkato@coh.org or

Rama Natarajan, rnatarajan@coh.org

Beckman Research Institute of the City of Hope
1500 East Duarte Road,
Duarte, CA 91010
Tel: 626-256-4673, ext 62289, 63996
Fax: 626-301-8136

Supplementary Figure 1

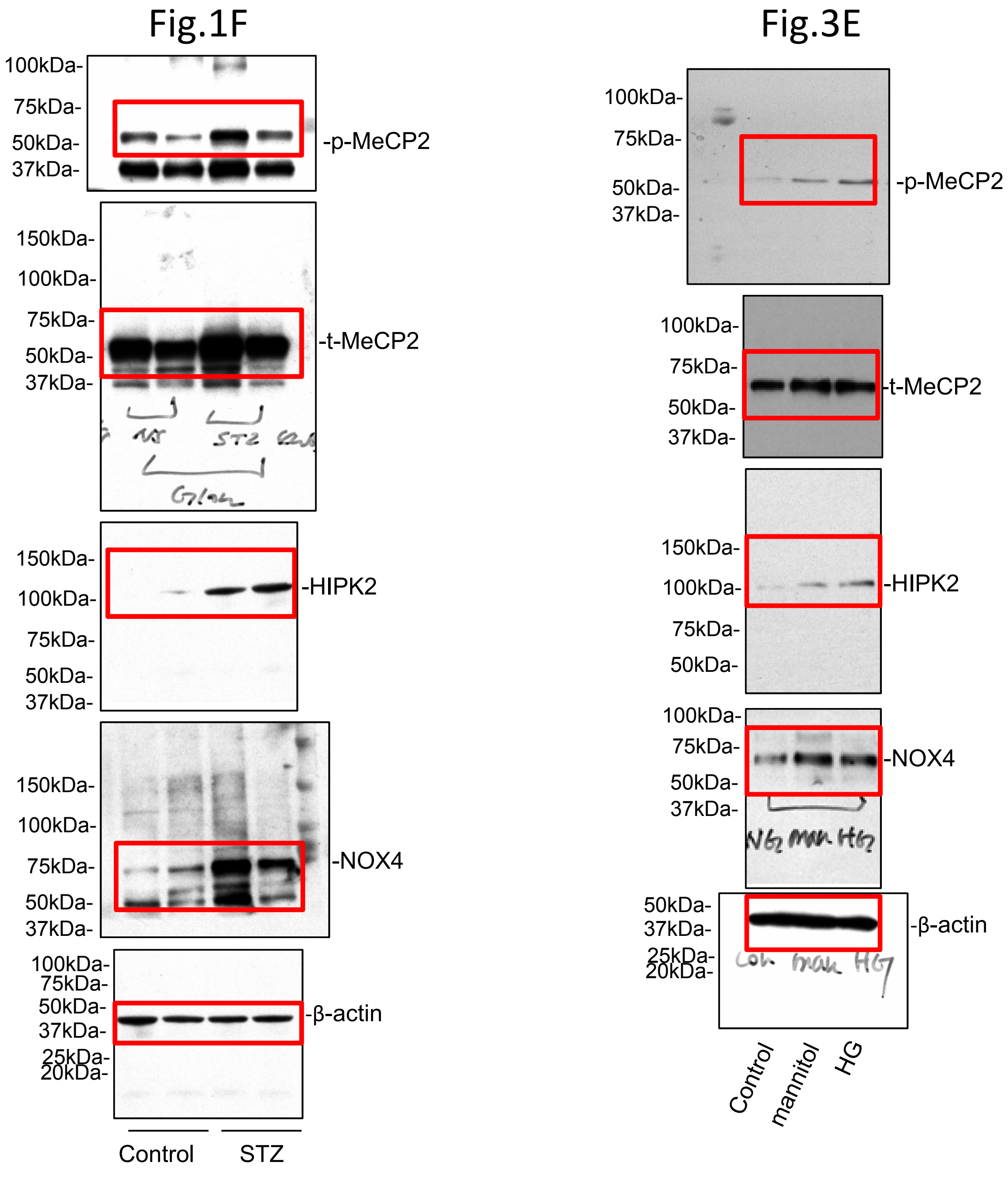

Supplementary Figure 1. Wider (uncropped) scans of blots (Fig.1F & Fig.3E).

Supplementary Figure 2

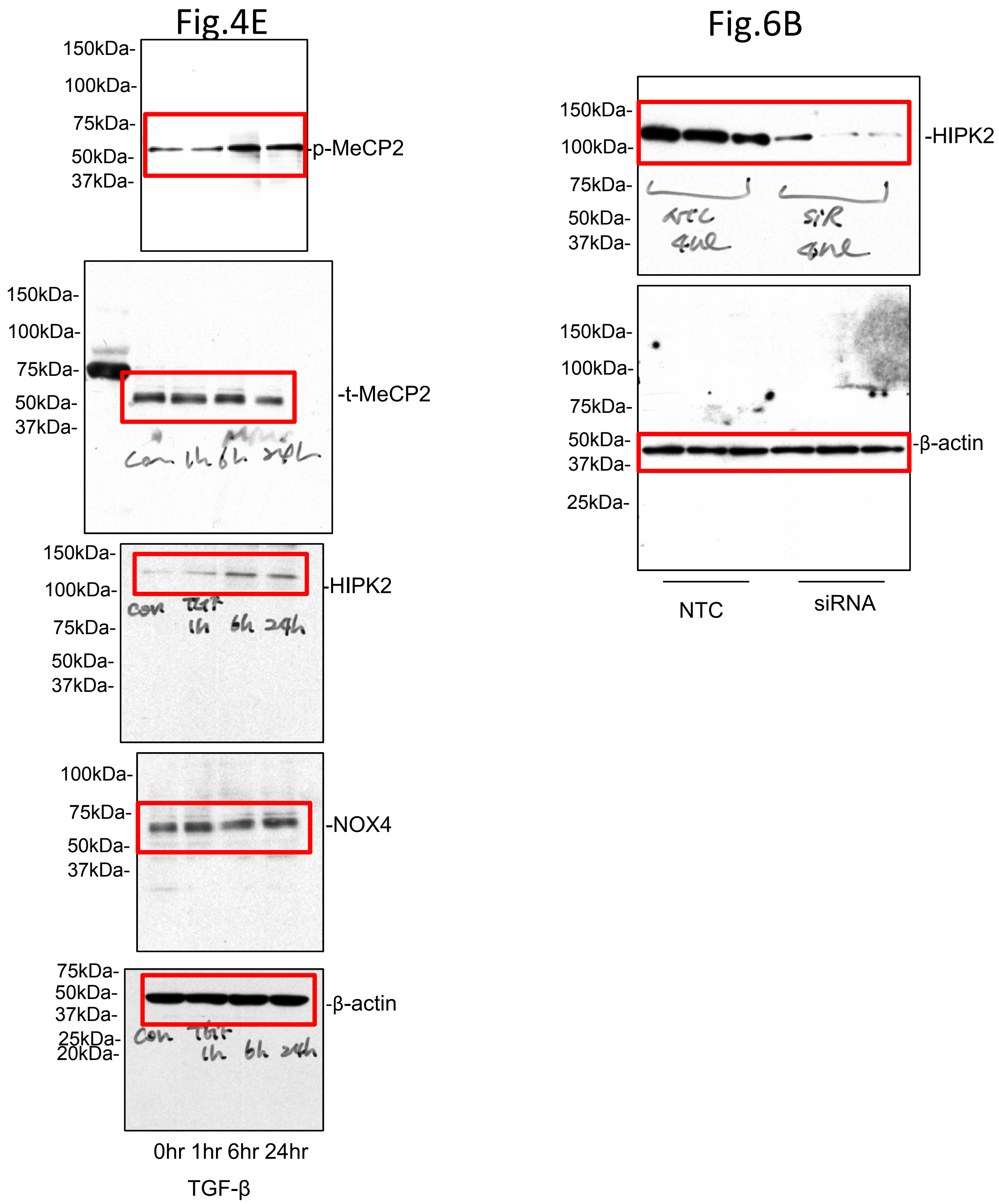

Supplementary Figure 2. Wider (uncropped) scans of blots (Fig.4E & Fig.6B).

Supplementary Figure 3

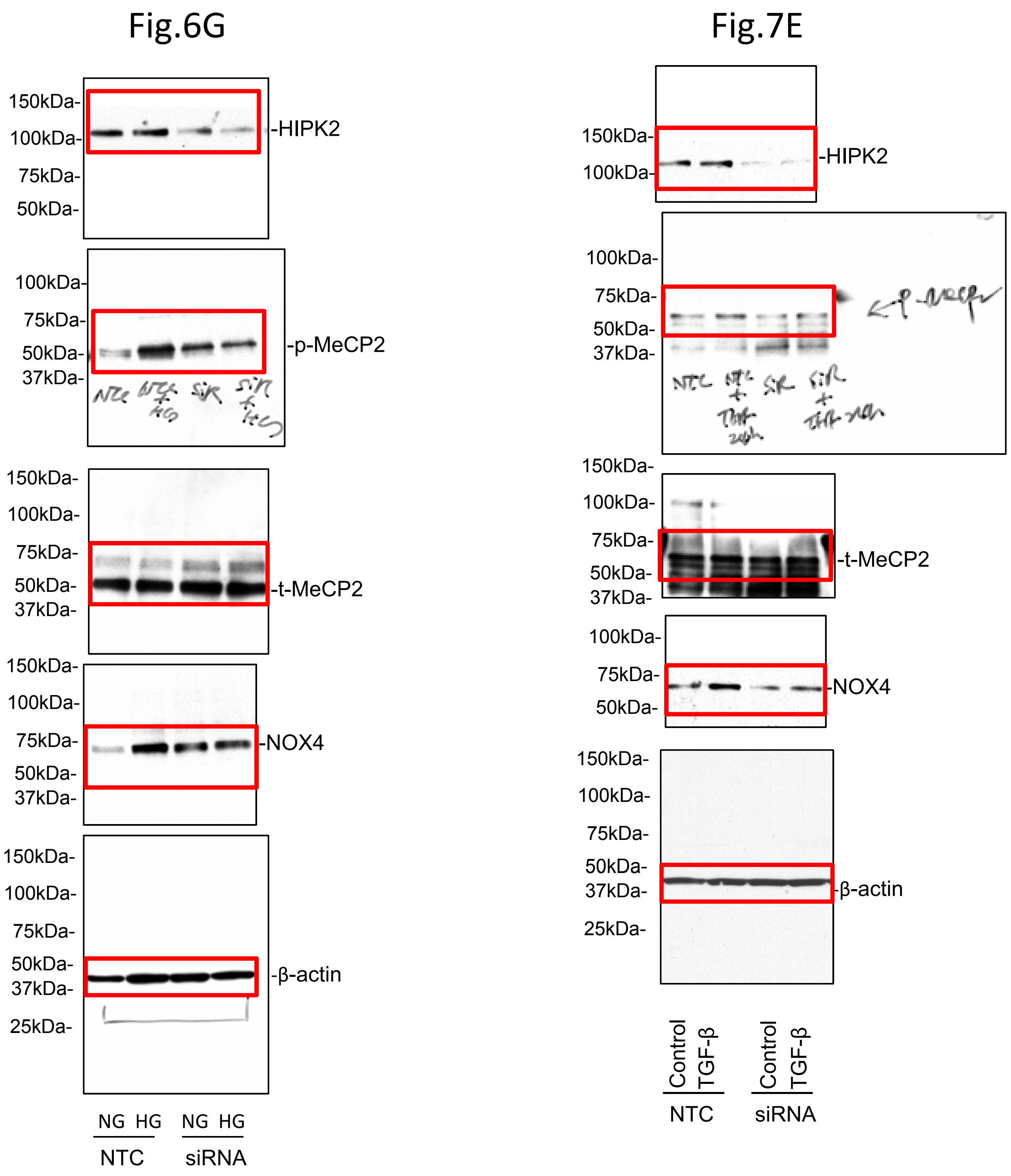

Supplementary Figure 3. Wider (uncropped) scans of blots (Fig.6G & Fig.7E).
